# Molecular mechanisms of protease precursor autoprocessing of RNA viruses: a comprehensive review

**DOI:** 10.1186/s12985-026-03119-z

**Published:** 2026-03-04

**Authors:** János András Mótyán, Mária Golda, Mohamed Mahdi, Nashaat T. Nashed, John M. Louis, József Tőzsér

**Affiliations:** 1 Department of Biochemistry and Molecular Biology, Faculty of Medicine, University of Debrecen, Debrecen, Hungary; 2 Laboratory of Chemical Physics, National Institute of Diabetes and Digestive and Kidney Diseases, National Institutes of Health, Bethesda, MD 20892-0520 USA

**Keywords:** RNA virus, Protease, Protease precursor, Autoprocessing, Self-cleavage, Polyprotein, HIV-1, SARS-CoV-2

## Abstract

Many viruses express their proteins in the form of large polyproteins comprising structural and non-structural (e.g. enzymatic) units that are released from the precursor through ordered proteolysis. Proteolytic processing of polyproteins is an indispensable regulatory step for virus maturation and replication that is carried out by the virus-encoded and/or cellular proteases. The activity of a viral protease that is expressed as a part of a polyprotein is controlled in part by the self-cleavage (autoprocessing) from the precursor. The mechanism of protease precursor processing has been established at the molecular level for various RNA virus proteases, including human immunodeficiency virus (HIV) and severe acute respiratory syndrome coronavirus 2 (SARS-CoV-2). Both viral protease precursors are processed *via* intra- (*in cis*) and intermolecular (*in trans*) cleavages at the N- and C-termini, respectively, yielding the mature enzyme. The remarkably similar activation mechanisms of HIV and SARS-CoV-2 PRs suggest that other viral proteases are activated similarly. In this review, we provide a detailed overview on the protease precursor autoprocessing mechanism of HIV-1 and SARS-CoV-2 proteases and compare those to the activation mechanism of non-viral proteases from their zymogens. Also, we review the activation mechanism of other ss(+)RNA viruses that utilize the polyprotein pathway for their replication. Based on such comparison, it appears that the protease activation mechanisms of most enveloped ss(+)RNA viruses from their precursors share many common features, although they do not correlate directly with the evolutionary relationships, the presence or absence of viral envelope or the catalytic mechanism of the viral protease.

## Introduction

### Viral polyproteins and viral proteases

**Fig. 1:**
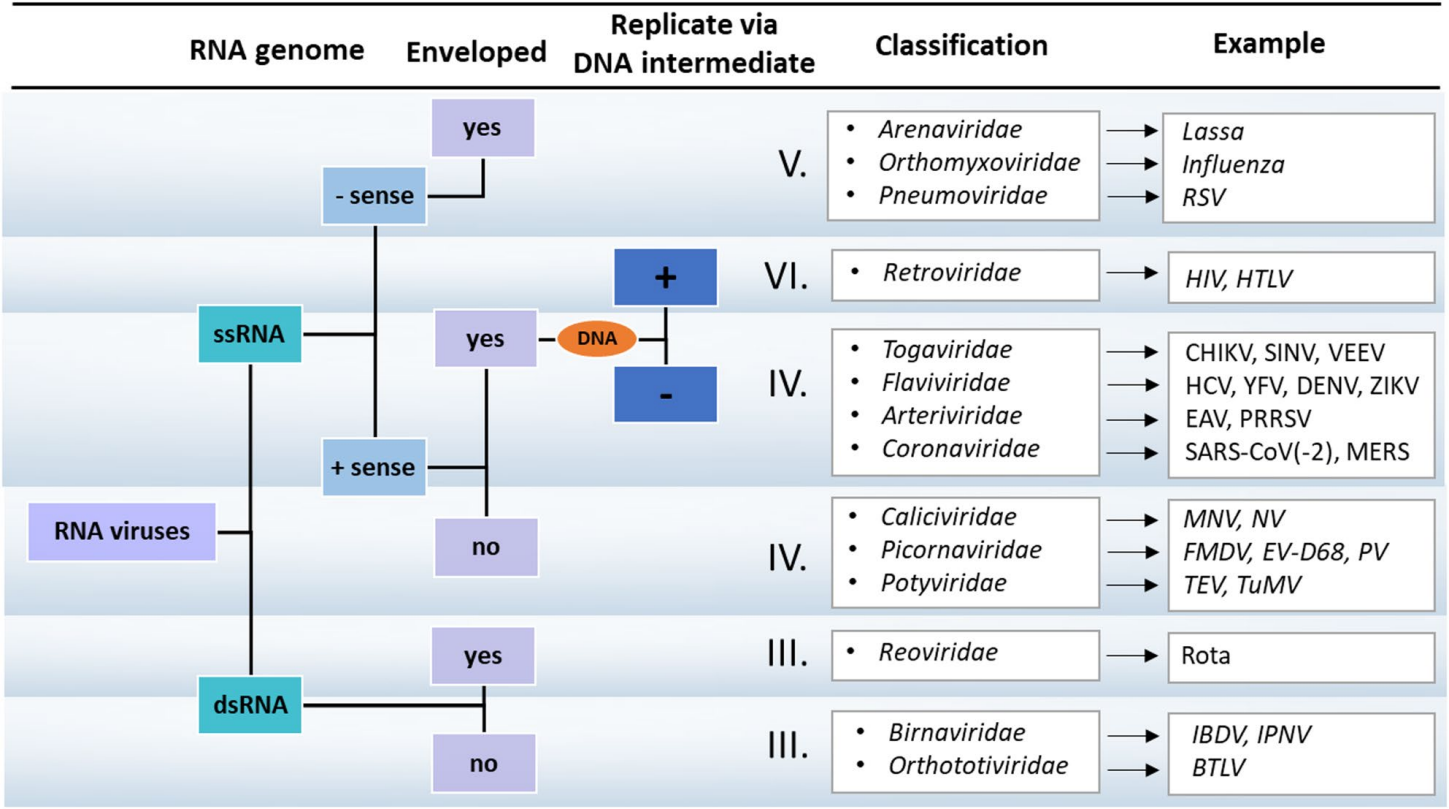
Classification of RNA viruses. The classification of RNA viruses is shown based on their genome and replication strategy (Baltimore classification), most important families and representative examples are shown

Viruses are classified based on their morphology, chemical composition and the mode of replication (Fig. 1). The genomic DNA or RNA is either single (ss) or double stranded (ds), and the single stranded genomes can be either positive or negative sense (ss(+) and ss(-), respectively). The genomic RNA of the ss(+)RNA viruses (also known as sense-strand) can directly serve as mRNA for the synthesis of viral proteins by the host cell’s translation machinery. The genome of ss(-)RNA viruses acts as complementary strand, also referred to as antisense strand, from which a positive-sense strand is synthesized by the viral RNA-dependent RNA polymerase [50]. Due to the differences of their compositions, viruses adopt different pathways for protein synthesis. One pathway involves the expression of the proteins as parts of polyproteins. In many cases, the large precursor polyprotein comprises one or more viral proteases (PRs) that subsequently process the polyprotein *via* proteolysis at discrete sites, although, host proteases may also contribute to polyprotein processing [141]. During such processing, the polyproteins are precisely cleaved only at specific sites, releasing the functional units (structural proteins and enzymes) from the large precursor, that ensures proper timing for replication, assembly as well as maturation. The regulation of polyprotein processing is not limited to the cleavage sites, other factors also contribute to the control of proteolysis, e.g. (i) some proteases need a cofactor, (ii) the precursor and mature proteins might exhibit differences in their activity or localization, (iii) the sequences of the recognition sites influence the kinetics of proteolytic processing, and thus determine in part the order of the cleavages at the individual sites, and (iv) the efficacy of precursor processing is dependent in many cases on the conformational accessibility of the cleavage sites. As discussed later, both precursor and mature forms of the viral PRs contribute to the order of proteolysis of the large polyproteins, including intra- and intermolecular cleavages.

Unlike ss(+)RNA and some DNA viruses, the genomes of other DNA viruses and most ss(-)RNA as well as most dsRNA viruses do not code for a known viral PR [111]. The virus-encoded PRs are structurally diverse, but they are well regulated with high sequence specificity for polyprotein processing [40, 82, 160, 173, 201]. PRs make a significant contribution to the replication, at different stages of the viral life-cycle [40]. Their function includes not only the cleavage of the viral polyproteins at discrete sites but also performing the cleavages in a proper sequential order, which is essential for virion assembly and maturation, as reviewed previously [89, 176]. There are multiple factors that influence the proteolytic processing, but it is not fully understood how the order of the cleavages is determined by the various factors. Nevertheless, it is known that imprecise cleavages and order lead to aberrant assembly and production of non-infective immature virions [89, 135, 189]. The significance of polyprotein processing lies not only in releasing the protein domains from the precursors, but also in serving as a functional control in time and space, as the precursor and mature forms of the proteins can differ in their functional characteristics, such as catalytic activity and the cleavage site sequence specificity. The cleavages are not limited to the viral proteins knowing that the host proteins are susceptible to inactivation by proteolysis and thereby contributing to viable replication. For example, viruses may evade the host immune reactions by manipulating cellular processes, either by proteolysis-dependent or -independent manner [24, 30, 85, 115, 142, 171, 177]. For example, HIV-1 PR can downregulate the level of RIG-I pattern recognition receptor protein that can activate antiviral responses upon recognizing viral ssRNAs [157]. In addition, receptor-interacting protein kinases (RIPK1 and RIPK2), that are activated in response to viral infection, are also cleaved by HIV-1 PR, causing their inactivation [182].

### Processing of polyproteins and activation of viral proteases

The initially synthesized polyproteins of the viruses whose genomes encode one or more PRs [40, 110] are considered as the full-length PR precursors. Self-cleavage is defined as autoprocessing, catalysed by the PR embedded within the polyprotein, resulting in the liberation of a fully active mature PR [79, 125]. Despite belonging to different structural classes and utilizing different catalytic mechanisms, a common feature of the virus-encoded PRs is their high substrate specificity [6, 15, 110, 111, 160]. Autoprocessing of the polyproteins may be carried out by either unimolecular or bimolecular reactions, i.e., intra- and intermolecular cleavages, respectively, to liberate the mature PR. The two reactions are distinguishable by their kinetic behaviour, irrespective to the involvement of more than one polypeptide chains, such as in the case of homodimerization (e.g. HIV-1 and SARS-CoV-2 main protease, MPro) or the contribution of a cofactor. An intramolecular reaction (*in cis* cleavage) displays first-order kinetics in protein concentration of the reacting protein species. The order of the reaction can be determined by observing a linear relationship between the initial rate of conversion and the protein concentration such as in the case of HIV-1 PR where the monomer lacks catalytic activity and the monomer-dimer equilibrium constant is well below the protein concentration in the model system used [102] or a constant percent conversion of the initial protein concentration to products in a defined amount of time at various initial protein concentrations, i.e. dilution-insensitive, as in the case of SARS-CoV-2 MPro [2] where the reaction takes place mainly from the monomer. In contrast, the intermolecular reaction (*in trans* cleavage) displays second-order kinetics in protein concentration, i.e. one molecule cleaves another molecule. In this case, the order of the reaction is determined by observing a linear relationship between the rate of cleavage and the concentration of the fully active enzyme, or between the initial rate of the reaction and the square of the protein concentration. It should be noted that observing a dilution-sensitive experiment would rule out only a unimolecular reaction but does not confirm a second-order reaction as the reaction may be of higher order [40, 102].

### Methods for the investigation of protease autoprocessing

The full-length polyproteins are not suitable for studying precursor processing in vitro because of their large size and the presence of multiple natural cleavage sites. Autoprocessing reactions are typically studied using model polyproteins, which mimic protease precursors. Invariably, they contain the PR domain flanked by native cleavage sites at one or both termini, and in many cases, native protein domains and/or fusion tags. For example, model HIV-1 precursors contained the full-length or truncated trans-frame region (TFR) and/or p6* domain at the N-terminus and a truncated reverse transcriptase (RT) domain at the C-terminus of the PR [127, 148]. In some cases, fusion tags were also attached to HIV-1 PR precursor mimetics, such as maltose binding protein (MBP) [102, 170], mCherry and enhanced green fluorescent protein (EGFP) [73], or glutathione S-transferase (GST) and Flag [68, 170]. Similar model precursors have been designed for SARS-CoV-2 MPro [Nashed, Kneller et al., 2022] [2, 118, 124] and papain-like protease (PLpro) [128]. A model precursor of SARS-CoV-2 MPro encompassed MPro and its N- and C-terminal cleavage sites (nsp4/nsp5 and nsp5/nsp6, respectively) [2], and similar mini-precursors were prepared for MPro of SARS-CoV and Middle East respiratory syndrome coronavirus (MERS) [28, 72, 117]. The cleavage assays performed by using model proteins permit monitoring the kinetics of precursor processing in vitro or in cell culture-based experiments. Tagging the N- and/or C-termini of a model precursor enables following the reactions at both termini simultaneously as well as the kinetic order of each of these processes.

Besides investigation of model precursors by in vitro activity assays, experimental approaches such as X-ray crystallography, nuclear magnetic resonance (NMR) and cryogenic electron microscopy (cryo-EM) (for example HIV-1: 7SJX.pdb [60] and SARS-CoV-2: 8EKE.pdb [118]) have been used to gain insights into events leading to the processing reaction of polyproteins and PR precursors [167]. Structural studies can provide information about the conformations that are unique to the precursor, intermediates in its conversion and the mature forms. Although, polyprotein processing is common to many virus families, sufficiently detailed structural information is available only for a limited number of cases, such as HIV-1 PR’s [27, 74, 107, 147, 168], SARS-CoV [66] and SARS-CoV-2 MPro [95, 123] being the most thoroughly studied. Furthermore, structure determinations provided insights into the conformational characteristics of PR precursors or proteolytic events such as intra- and/or intermolecular interactions. In addition, direct or indirect lines of evidence have been reported for the PR of the flavivirus Zika virus (ZIKV) [136] and for alphavirus PRs, such as that of Sindbis virus (SINV) [153] and Venezuelan equine encephalitis virus (VEEV) [34, 65].

Attempts to suggest a mechanism of autoprocessing reaction from crystallographic X-ray structure can be misleading as X-ray structures are static conformations. The observation of an N- or a C-terminus bound to the active site of an X-ray structure is no direct evidence of a mode of reaction as both termini are substrates and products for the enzyme, and thus, expected to bind to the active site. Also, any conclusion about the processing reaction, i.e., *in cis* or *in trans* cleavage, based on the X-structure of the mature enzyme ignore the possibility of conformation changes that may occur upon the autoprocessing reaction, see for example the discussion below for HIV-1 PR. Alternatively, molecular dynamics can be utilized to investigate the conformational characteristics of the precursor and mature forms and provide insights into the dynamics and conformational rearrangements of the self-activation reactions [65, 86, 147]. The only direct evidence for the mechanism of autoprocessing reaction must come from a kinetic experiment as the order of the reaction reflects the composition of the transition state of the cleavage.

## Protease precursor processing of RNA viruses

### Enveloped ss(+)RNA viruses - Retroviral proteases - HIV-1 PR

**Fig. 2:**
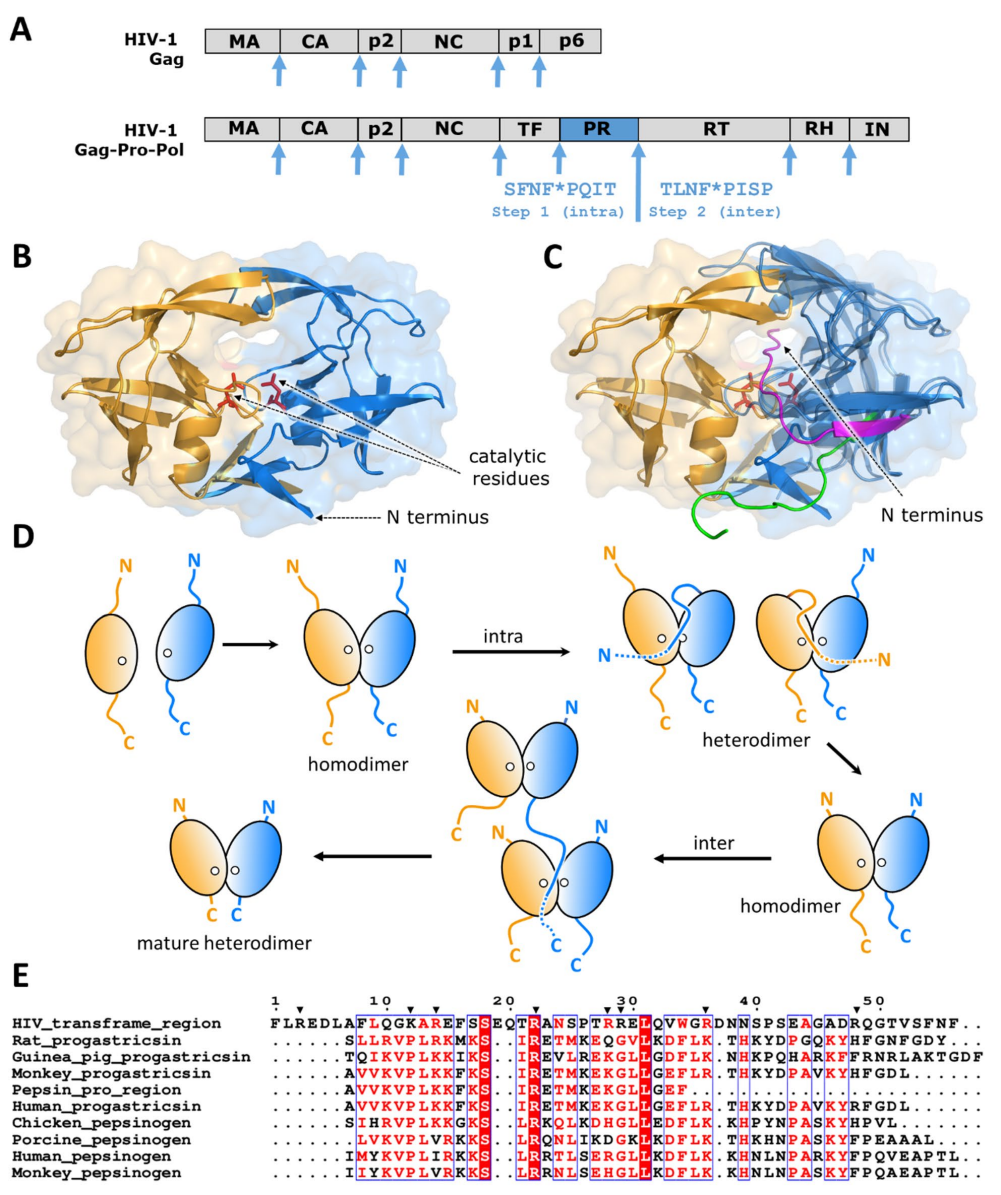
Structural representation of *in cis* N-terminal autoprocessing of HIV-1 PR. (**A**) Organization of HIV-1 Gag and Gag-Pro-Pol polyproteins. The PR is highlighted in blue along with the cleavage sites (arrows) and their sequences, the sequential order of cleavages is labeled. (**B**) Ribbon representation based of the crystal structure of mature HIV-1 PR (PDB ID: 5HVP) [48]. (**C**) The conformational states of the N-terminal cleavage site of the precursor are shown based on structures determined by NMR. N-terminal residues are shown in green and purple based on the solution NMR structure (PDB ID: 1Q9P) [74] and a model prepared from structural coordinates provided by Chun Tang [168]. Ribbon/surface representations of the PR subunits are shown in light orange and blue and the catalytic residues are shown as red sticks. (**D**) Schematic representation of protease precursor autoprocessing *via* dimerization from the viral polyprotein. Polypeptide chains corresponding to the PR precursor subunits are shown in blue and yellow. The aspartate residues at the active sites of the monomers are indicated by white circles, the catalytic conformation contributed by Asp-Thr-Gly triad is formed upon dimerization. Dashed lines represent the truncation of N and C-termini. (**E**) 56 residue long trans-frame region (TFR) upstream from HIV-1 PR aligned with the pro-sequences of several non-viral PRs [80]. Arrowheads show Lys and Arg residues of HIV-1 TFR. Sequence alignment was performed by using ESPript 3.0 online tool [143]

HIV, the causative agent of acquired immunodeficiency syndrome (AIDS) belongs to *Retroviridae* family of enveloped ss(+)RNA viruses (Fig. 1). Retroviruses are unique among RNA viruses as they replicate *via* a DNA intermediate. During their replication, their RNA genome is reverse transcribed to DNA that is subsequently integrated into the host cell’s DNA by the viral integrase. The integrated provirus then serves as a template for the synthesis of the new viral RNA and proteins [33]. HIV viruses utilize the polyprotein pathway for their replication; their genomes code for two large polyproteins, the Gag and the Gag-Pro-Pol, of which the larger Gag-Pro-Pol is synthesized *via* ribosomal frameshifting. Genomes of HIV-1 and HIV-2 also encode a single copy of an aspartic PR that is synthesized as a part of the Gag-Pro-Pol polyprotein (Fig. 2A). HIV PRs are homodimeric having one active site per dimer in which each of the subunit contributes half of the catalytic residues. HIV PRs autocatalyze their own cleavage for activation from the polyproteins (Fig. 2B), as described below.

The mechanism of HIV-1 PR’s activation has been studied extensively using model polyproteins that comprised the PR domain and flanking sequences that limit the number of the PR cleavage sites at the N and/or C-termini. Model polyproteins containing the trans-frame region (TFR) peptide (TFP) and/or p6^pol^ attached to the N-terminus of PR were found to undergo cleavage at the p6^pol^/PR site accompanied by a large increase in catalytic activity in a time-dependent manner. The reactions were monitored by following the appearance of the catalytic activity and the mature enzyme as well as the disappearance of the precursor. They display first-order kinetics in protein concentration indicating that the cleavage is an intramolecular reaction (Fig. 2D). A polyprotein containing the octapeptide (TFP) followed by p6^pol^ and the PR domain having the natural cleavage sites TFP/p6^pol^ and p6^pol^/PR (TFP-p6^pol^-PR) undergoes two intramolecular cleavage steps sequentially below pH 5 and a single cleavage at the N-terminus above pH 5 accompanied by a large increase in catalytic activity [103, 104]. Both TFP-p6^pol^-PR and p6^pol^-PR have low catalytic activities. Since the monomeric HIV-1 PR has half of the catalytic residues of the active site, dimer formation must occur prior to the intramolecular cleavage [102, 104–107]. In contrast, the PR containing the C-terminal ΔPol (Δ denotes truncated) sequence has catalytic activity comparable to that of the mature enzyme, and the C-terminal cleavage displays second-order kinetics in protein concentration, i.e. intermolecular reaction [190]. While the intramolecular cleavage at the N-terminus with native flanking sequence is a prerequisite to produce a fully active protease, other cleavages upstream from this site may occur intramolecularly in the full-length polyprotein in addition to the TFP/p6^pol^ cleavage [104, 110, 133–135].

The mechanism of activation of the homodimeric HIV-1 PR from the polyprotein precursor (Fig. 2D) is nearly identical to that for the activation of the single polypeptide chain of the non-viral aspartic proteases. The main difference is the requirement of the viral PR to form a homodimer and generate a competent active site (see the structure of the mature enzyme Fig. 2B and C). HIV-1 PR truncated at the N-terminus (residues 1–4) and the C-terminus (residues 96–99) exhibits a stable monomer fold spanning residues 10–90 like that of the single subunit of the wild-type dimer [74, 75]. An enzymatically inactive mini-precursor consisting of four residues of the trans-frame region fused to the N-terminus of the inactive PR domain is shown to form a small (3–5%) population of dimeric species by paramagnetic relaxation enhancement NMR [168]. Model polyprotein comprising the PR domain displays similar characteristics to the non-viral aspartic PR zymogen such as low enzymatic activity, binding inhibitors, and the intramolecular autocleavage at its N-terminus indicating that a fully dimeric polyprotein has a functional active site, see studies on TFP-p6^pol^-PR model HIV-1 polyprotein in the previous paragraph [104]. Another similarity is found in the amino acid sequence upstream from the viral PR domain: while the amino acid sequence upstream from the viral PR polyprotein, i.e. the 56 amino acid residues of TFP-p6^pol^, has little sequence homology to the positively charged pro-sequences of the non-viral aspartic PRs (11–14 Lys and Arg residues), it comprises 8 Lys and Arg residues (Fig. 2E). Given the facts that the liberation of the N-terminus of the mature PR is generated by autocatalytic reaction and the location of the N-terminus of the mature PR on the opposite side of the enzyme’s active site participating in an extended β-sheet (Fig. 2C), the cleavage at the N-terminus of HIV-1 PR must be accompanied by similar conformational changes to those observed in the non-viral enzymes to open the active site and make it available for substrates [81, 82, 95]. This conclusion is further supported by the study utilizing a model polyprotein having non-native sequences flanking the N-terminus of the PR [191]. In contrast to model polyproteins comprising native cleavage sites at the N-terminus, constructs having non-native cleavage sites (X_28_-PR and X_28_-ΔTFR-Ala-PR-Ala-ΔPol, where X_28_ is a random amino acid sequence) do not autoprocess at the N-terminus. They display enzymatic activities comparable to the mature enzyme and increased dimer dissociation constant. This indicates that a free terminal residue is not required to organize the active site for enzymatic activity, but it is required for stabilizing the dimer form of the enzyme. That raises the question: why does a polyprotein with native flanking sequence have low enzymatic activity? The only plausible answer is that the upstream sequence in the HIV-1 polyprotein is interacting with the active site residues and blocks the active site in a similar manner to that of the pro-sequence of the non-viral aspartic acid PRs [82]. This conclusion is further supported by the observed inhibition of the mature protease by the trans-frame peptide (TFP) and p6^pol^ containing its C-terminal residues [103, 131]. Recent studies have also indicated that specific amino acid substitutions in the TFR significantly impair virus infectivity [199].

### Enveloped ss(+)RNA viruses - Coronavirus proteases - SARS-CoV and SARS-CoV-2

**Fig. 3:**
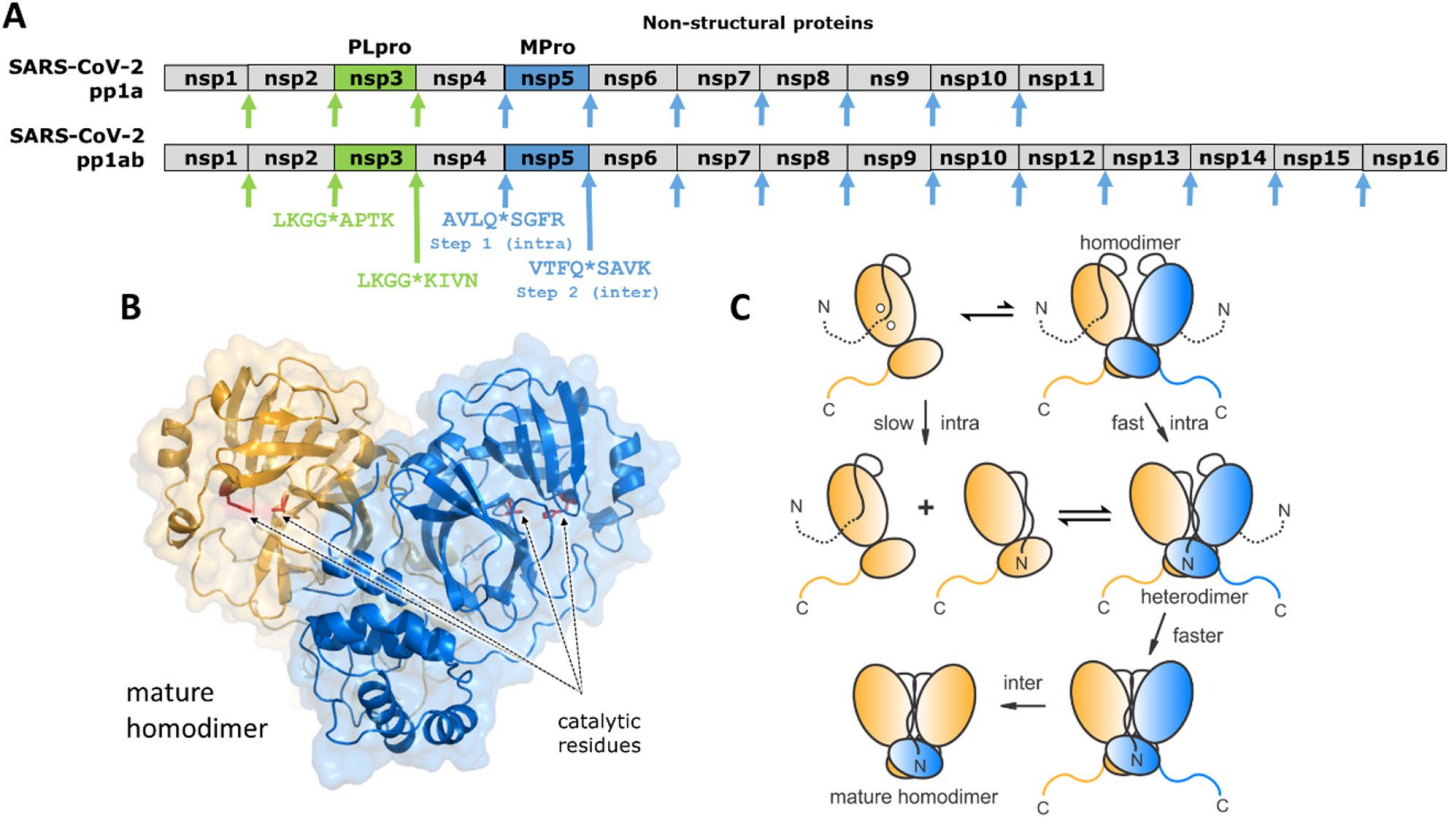
Steps in the autoprocessing of SARS-CoV-2 MPro. (**A**) Organization of pp1a and pp1ab SARS-CoV-2 polyproteins. PLpro (nsp3) and MPro (nsp5) are highlighted green and blue, respectively. Arrows pointing to the cleavage sites, and their sequences are colored accordingly. (**B**) Mature MPro crystal structure (PDB ID: 7JOY) [95]. The subunits are shown as surface/ribbon representations in light orange and blue. (**C**) Proposed mechanism of MPro autoprocessing. This figure part was reproduced from Fig. 7 of Aniana et al., [2] (http://creativecommons.org/licenses/by/4.0/) with recoloring of the MPro domains and additional labels in the legend. Catalytic (domains I and II) and helical (domain III) regions are shown as orange and blue ovals, respectively. Solid black lines denote the N-terminal residues (N-finger) of MPro. Dashed black and solid blue lines represent the truncated nsp4 and nsp6 regions flanking the N and C-termini of MPro, respectively. Catalytic dyad H41 and C145 residues are indicated as white circles in the top left monomer cartoon

SARS-CoV, SARS-CoV-2 and MERS are enveloped viruses having ss(+)RNA genome. They belong to Group IV of viruses and are classified into the *Betacoronavirus* genus of the *Coronaviridae* family (Fig. 1). The genomic organization of SARS-CoV and SARS-CoV-2 are similar and code for multiple non-structural proteins (nsps), two of them are cysteine PRs (Fig. 3A). Nsp3 is a papain-like PR (PLpro), while nsp5 is a 3-chymotrypsin-like PR (3CLpro) and referred to as the main protease (MPro). Both PRs play an important role in processing the viral polyproteins. MPro of SARS-CoV and SARS-CoV-2 share 96% sequence identity and differ only by 12 residues [96]. They are expressed embedded in polyproteins from which MPro autocatalyzes its own cleavage to form homodimer (Fig. 3B C). MPro consists of three domains: the chymotrypsin-like domain I and II contain the active site of the enzyme while domain III (helical domain) is involved in enhancing dimer stability. The two subunits form an asymmetric interface through their N-terminal residues (“N-finger”) and domain III [119]. Upon dimerization of MPro, the active site oxyanion loop is transitioned from a predominantly unwound inactive conformation (E) in the monomer to a wound active conformation (E*), which has been clearly demonstrated both for SARS-CoV and SARS-CoV-2 MPro [91, 120, 194].

Several studies attempted to examine the mechanism of self-maturation by crystallography; N- and C-terminal mutations and X-ray structure analyses resulted in differing conclusions for both SARS-CoV [28, 66, 100, 117] and SARS-CoV-2 [123]. Recently, model polyproteins of SARS-CoV-2 containing native cleavage site at both termini has revealed sequential order of the cleavages during autoactivation. The first cleavage is at the N-terminus of MPro and its percent cleavage in a window of time is constant and independent from the initial protein concentration of model polyproteins, which indicated that the N-terminal cleavage displays first-order kinetics and the reaction is intramolecular (Fig. 3C) [2].

While the mechanism of activation of MPro from the polyprotein differs from the mechanism of activation of the eukaryotic chymotrypsin family of enzymes, the properties of the model polyprotein and the eukaryotic zymogens as well as the structural changes that are observed after cleaving a peptide bond are strikingly similar. The zymogens of the eukaryotic chymotrypsin family of enzymes are single polypeptide chains having low enzymatic activities, bind inhibitors and small molecules indicating that there are two conformers of the zymogen, an active conformation E* and inactive conformer E [25]. Similar observations were made in several model dimer polyproteins. Model polyprotein precursors in which key residues such as E290 and R298 in the dimer interface are mutated or the helical domain is completely deleted and containing native flanking sequences at the N- and C-termini have been studied. In all cases, the model polyproteins were fully processed at their N-termini both in vivo and in vitro, indicating that the PR is folded and having enzymatic activity, and bind inhibitors that are designed for the mature MPro [91, 92, 119, 120]. Model protein of MPro carrying the E290A and R298A (MPro^M^) mutations and containing a native flanking sequence at the N-terminus binds the inhibitor GC373 and accelerate the N-terminal cleavage in vitro [2]. Such inhibitor-induced acceleration of activity was also observed for the MPro precursor in enzyme kinetic assays [Novotny et al., 2025]. Mature MPro^M^ displays low enzymatic activity from a small population of the dimer, but the catalytic activity increases significantly upon the addition of an optimal concentration of a known inhibitor of MPro^WT^. Inhibitor binding to MPro^M^ stabilizes the dimer form and results in the formation of an increased population of dimer molecules in which one of the active sites is occupied with the inhibitor, and the other is in the proper conformation and available for catalytic activity and thereby resulting in an overall increased catalytic activity [119]. The mature MPro lacking the dimerization domain III (MPro^1–199^) displays very low enzymatic activity only from the monomeric form [120]. In contrast, the product of the N-terminal cleavage of a model polyprotein containing the C-terminal native flanking sequence or purification tag has comparable enzymatic activity to the mature enzyme. The C-terminal flanking sequence is processed *via* intermolecular reaction mediated by the action of a mature wild-type MPro [2].

The mechanism of activation of the chymotrypsin family of enzymes and the structural changes associated with the generation of a new N-terminus have been reviewed [81]. The eukaryotic chymotrypsin family of enzyme are activated by intermolecular cleavage. For example, the activation of chymotrypsinogen is initiated by cleaving the peptide bond Arg15-Ile16 catalysed by trypsin or enterokinase. Comparing the structure of chymotrypsinogen and α-chymotrypsin shows that the newly generate amino terminus Ile16 forms an ion pair with Asp194 triggering the formation of the active enzyme by reorganization of a disordered loop (residues 186–194) in the zymogen to form the oxyanion hole and makes the active site accessible to the substrate. Similarly, comparing the room temperature X-ray structures of monomeric MPro^1–199^ with and without the inhibitor GC373 show the protein has a native like fold similar to that of the wild type with the active site dyad Cys145-His41. However, unlike the native mature MPro dimer, the structure of MPro^1–199^ shows that the oxyanion loop in an unwound inactive conformation E*, whereas the structure of MPro^1–199^-GC373 complex display an oxyanion conformation in the active conformation (E) like that of the native dimer [120]. The newly generated N-terminal Ser1 of MPro plays a critical role in stabilizing the active E*-state like that observed in eukaryotic chymotrypsin family of enzymes. High resolution X-ray and neutron crystal structures of the native dimer show the protonated α-amino group of the N-terminal Ser1 of one subunit forms an ion pair with the carboxylate side chain of Glu166 of the other subunit of the homodimer, i.e. an inter-subunit interactions which stabilizes the active conformation of the active site [29, 88, 95]. That intermolecular ionic pairing requires dimer formation for stabilizing the active conformation of the oxyanion loop and to unmask the wild-type activity.

Besides MPro, genomes of coronaviruses code for one or two papain-like PR (PLpro), as well (Fig. 3A). Members of *Alphacoronavirus* genus have two PLpro (PL1pro and PL2pro), while many of *Betacoronavirus* genus have only a single PLpro, such as SARS-CoV, SARS-CoV-2 and MERS. PLpro is a C-terminal domain of nsp3 that is a multifunctional cysteine PR. The characteristics of PLpro are well-reviewed in the literature [4, 87, 98, 109, 178, 192, 196]. SARS-CoV PLpro is active in its monomeric form and one of its main functions is the proteolytic processing of pp1a and pp1ab viral polyproteins at three specific sites to release nsp1, nsp2, and nsp3 [57]. PLpro recognizes specific cleavage sites with the consensus sequence LXGG↓XX and cleaves the polyprotein to release itself and other non-structural proteins. This is supported by studies showing that PLpro can efficiently process its own N- and C-terminal cleavage sites during polyprotein processing [10]. These sites are susceptible for cleavage *in trans* [57]. SARS-CoV-2 PLpro was suggested to undergo autoproteolysis [128], based on the ~ 83% shared sequence identity with highly conserved catalytic triads and similar “thumb-palm-fingers” structures between SARS-CoV and SARS-CoV-2 PLpro [126]. Precursor processing of PLpro was studied recently in vitro by dose-dependent transfection and recombinant reporter substrates [99]. The studies revealed that the occurrence of the cleavage at nsp2/nsp3 cleavage site at low protein concentration, while the cleavage at nsp3/nsp4 junction was found to require high protein dosage, indicating concentration-dependence, i.e. processing of nsp3/nsp4 cleavage site *in trans*. Intermolecular nature of the cleavage at this site was found to be determined by membrane association of nsp3 as well as its domain rearrangements. In contrast, results suggest that cleavage of nsp2/nsp3 junction can occur both *in cis* and *in trans* [99].

### Enveloped ss(+)RNA viruses - Flavivirus proteases

Dengue virus (DENV), Yellow fever virus (YFV), West Nile virus (WNV) and Zika virus (ZIKV) belong to the *Flavivirus* genus of the *Flaviviridae* family that have ss(+)RNA genome (Fig. 1). They are mosquito-borne viruses that are known to have public health implications [19], for example, ZIKV infection might be associated with congenital foetal abnormalities such as microcephaly [184].

**Fig. 4:**
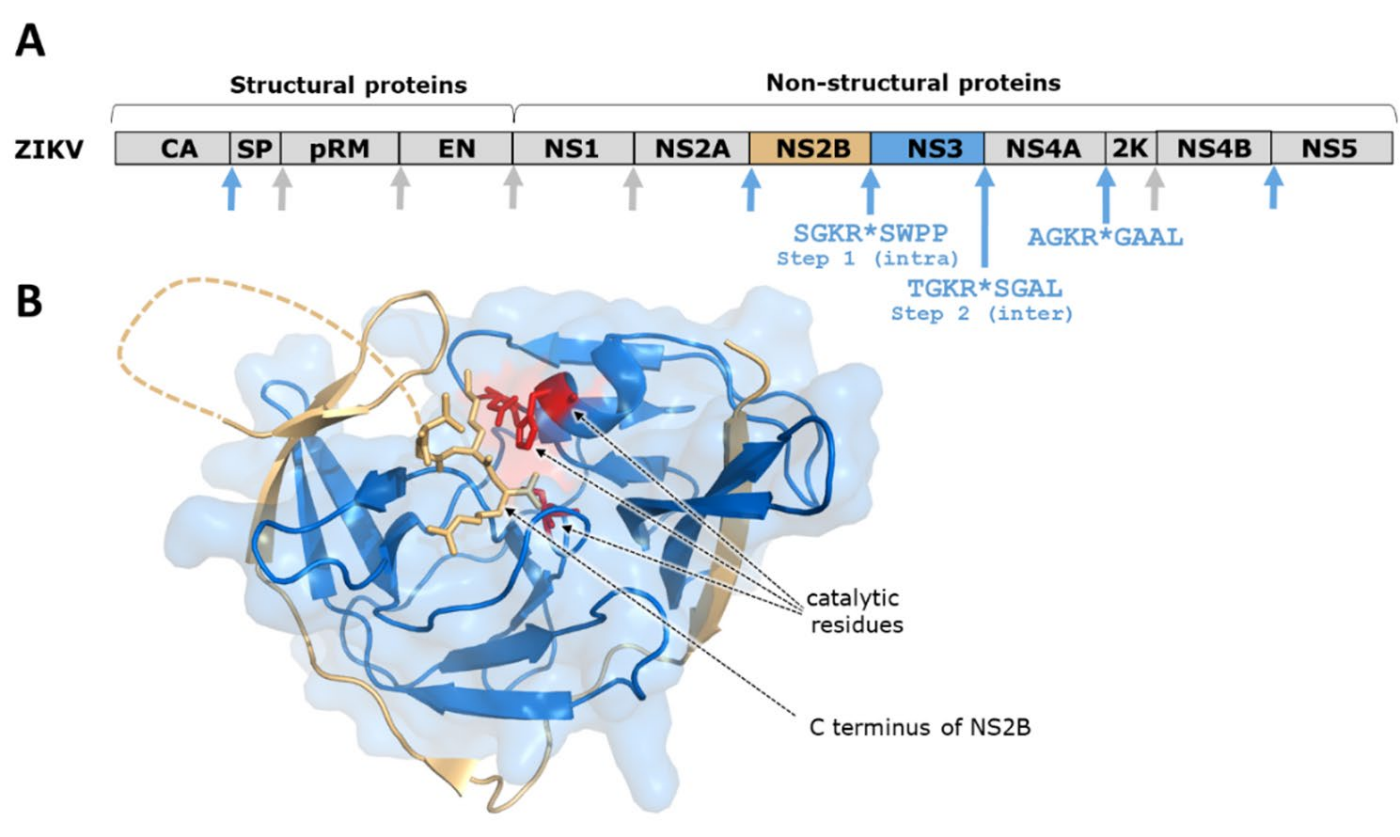
Structure of NS2B-NS3 protease of Zika virus. (**A**) Organization of ZIKV polyprotein. NS3 PR is highlighted in blue along with the cleavage sites (arrows) and their sequences. The cleavage sites of furin and other host PRs are indicated by grey arrows [140]. prM: pre-membrane, EN: envelope. (**B**) Ribbon representation of NS2B-NS3 (PDB ID: 5GJ4) [136]. Surface representation of NS3 PR domain in light blue. The NS2B domain is shown in light orange and the residues of the catalytic triad (Ser135, His51 and Asp75) by red sticks. The C-terminal residues of NS2B bound to the active site of NS3 PR are also shown as sticks. The dashed light orange line indicates a region that is not visible in the crystal structure

The RNA genomes of several viruses of *Flavivirus* genus are well characterized and have similar structure [183]. The genome of a virus encodes a single open reading frame for a large polyprotein comprising structural and non-structural proteins (NS1-5) (Fig. 4A). Of the non-structural proteins only NS3 possesses proteolytic activity, a chymotrypsin-like serine PR at the N-terminus of NS3. A transmembrane protein, NS2B, is required for its enzymatic activity and for anchoring NS3 to the endoplasmic reticulum [200]. NS2B and NS3 form a complex that is responsible for polyprotein processing, including cleavages at NS2A/NS2B and NS2B/NS3 sites. The in vitro processing of DENV polyprotein, comprising 101–218 residues of NS2A, all NS2B and the first 600 residues of NS3, showed that the cleavage at the NS2A/NS2B site preceded the cleavage at the NS2B/NS3 site [139]. This study provided indirect evidence that support *cis* cleavages at both sites. Similar results were observed for polyprotein constructs of YFV [26]. NS2B consists of approximately 130 residues and contains three hydrophobic domains believed to be transmembrane and a central hydrophilic domain of only 40–50 residues required as a cofactor (NS2B_CF_) for catalytic activity of NS3 protease [183]. Several studies have examined the cleavage at the NS2B/NS3 in detail by tethering NS2B_CF_ to NS3 or only to the protease domain of about 180 amino acid residues of NS3 using a flexible linker and mutation at NS2B/NS3 cleavage site [13, 136, 181]. The results of these studies provided further indirect evidence for a *cis* cleavage at the NS2B/NS3 site and indicating that cleavage at the N-terminus of NS3 is not required to organize the active site into catalytically active conformation unlike the eukaryotic chymotrypsin family of enzymes and MPro of SARS-CoV and SARS-CoV2 (see above). This appears to be more like the bacterial chymotrypsin-like α-lytic protease where the active site of the proenzyme has identical conformation to that of the mature enzyme [81]. Investigation of DENV NS2B/NS3 showed that the catalytically inactive form of the protein lacks self-processing, suggesting that the NS2B/NS3 cleavage occurs exclusively *in cis*. Nevertheless, co-incubation of active and inactive forms of NS2B/NS3/NS4A revealed that the NS3/NS4A junction is also cleaved intramolecularly [35]. NS3 contains an unexpected internal cleavage site, as well [169], which is also cleaved strictly *in cis*. Interestingly, a mutation of this cleavage site (G459L) was found to abolish cleavage at internal NS3 junction, causing *trans*-dominant suppression of the wild-type virus’ replication, an effect driven by the precursor accumulation rather than by general loss of protease activity. Therefore, inhibiting precursor processing of susceptible viruses is considered to be a promising strategy because the accumulation of uncleaved precursors that act as *trans*-dominant inhibitors can block the replication [35]. The inhibition of protease precursor processing is discussed in Sect. 3.

The X-ray structures for tethered and untethered NS2B_CF_-NS3 complexes have been reported for ZIKV (Fig. 4B) [97, 136, 200]. The structures are very similar. They show that the N-terminal 18 residues (49–67 of ZIKV) of NS2B_CF_ support the folding of NS3 by forming a β-strand inserted into the protease domain and C-terminal residues 68 to 96 forms a β-hairpin to generate the S2 and S3 pockets in the substrate binding site of NS3. The involvement of the N-terminal residues of NS2B in the folding of NS3, possibly as a chaperon, suggests that the cleavage at NS2A/NS2B which precedes the cleavage at NS2B/NS3 site is required to organize and stabilize the active site conformation. Based on the data discussed above, it is unclear whether the observed activity at the mutated NS2B/NS3 cleavage sites is an artifact of using the combination of NS2B_CF_ and the flexible linker. It is possible for the flexible linker to allow the small fragment NS2B_CF_ to assume the proper interaction with NS3 to produce a functional active site. In contrast, anchoring the full-length NS2B to the endoplasmic reticulum may result in a more rigid conformation that requires the cleavage at the N-terminus of NS3 to allow the proper interaction between NS2B and NS3 to produce a functional active site. In addition, the crystal structure of the helicase domain of YFV suggested that the cleavage at NS3/NS4A junction occurs *in trans*. The C-terminus appeared to be inaccessible for intramolecular cleavage and cannot bind to the active site without substantial structural rearrangement of either the PR or the helicase (PDB ID: 1YKS) [193].

**Fig. 5:**
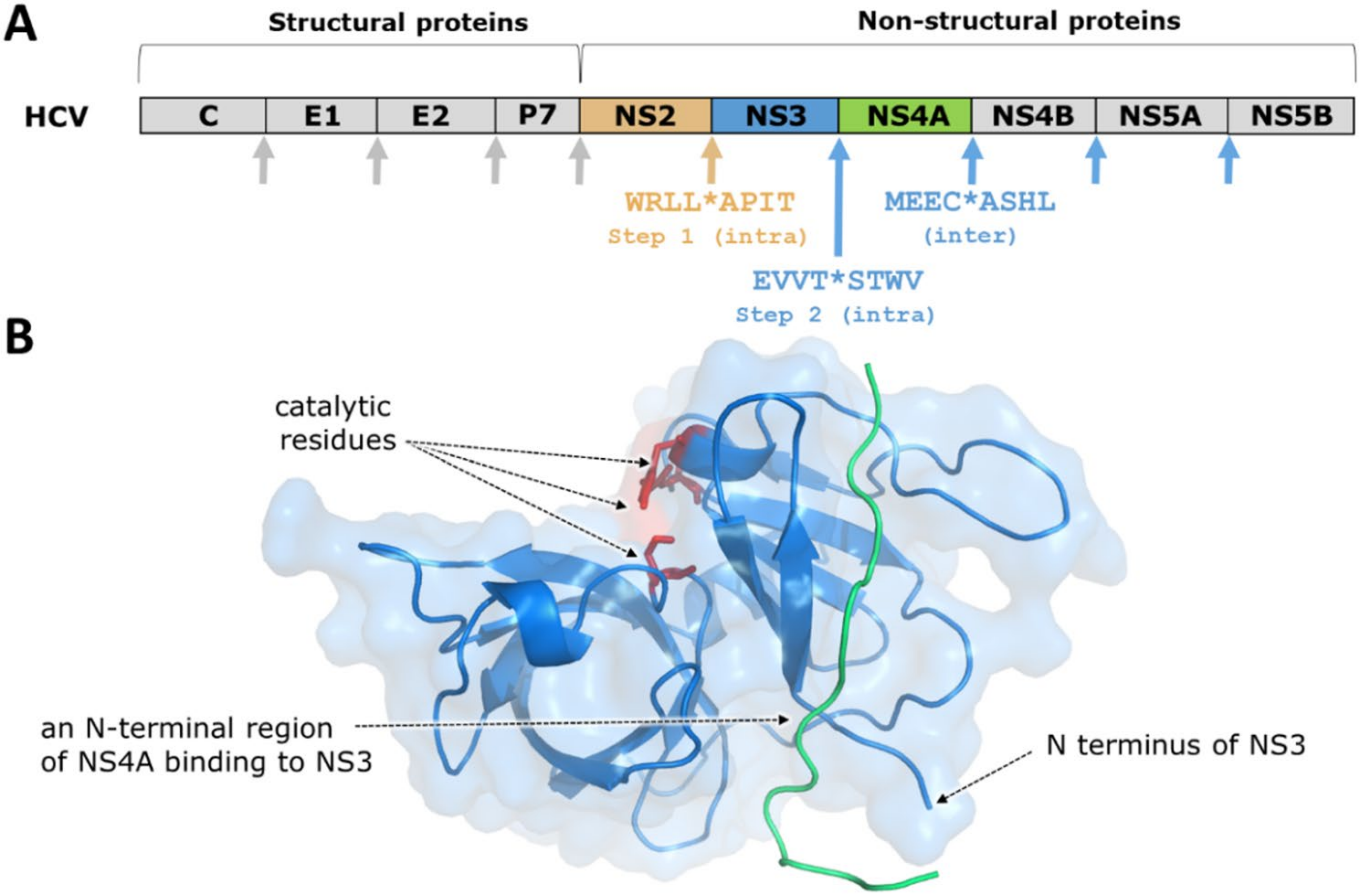
Structure of NS3 protease of Hepatitis C virus complexed with a short N-terminal peptide of its cofactor NS4A. (**A**) Organization of HCV polyprotein. The NS2 containing a cysteine PR, NS3 containing a serine protease domain and the NS4A cofactor peptide are shown in yellow, blue and green, respectively. Cleavage site sequences are colored accordingly. Cleavage sites of NS2 and NS3 are indicated by yellow and blue arrows, respectively. Cleavage sites of cellular signal peptidases are indicated by grey arrows. Defined cleavage sites are based on the reference [122]. C, E1 and E2 denote the core protein and the envelope glycoproteins E1 and E2, respectively. (**B**) X-ray structure of HCV NS3 PR complexed with NS4A cofactor peptide (PDB ID: 1A1R) [83]. The NS3 PR domain is shown by surface representation in light blue and the NS4A cofactor is green. The catalytic serine, histidine and aspartate residues are shown as red sticks

Most Flaviviruses seem to use the same mechanism for processing of PR precursor, the NS2B–NS3 cleaves at the NS2B/NS3 junction *in cis* and at the NS3/NS4A site *in trans*, like as been described for YFV [26], WNV [13] and DENV [139]. Interestingly, hepatitis C virus (HCV) that belongs to the *Hepacivirus* genus of *Flaviviridae* virus family exhibits different processing pathway than other flaviviruses such as ZIKV. Two HCV PRs are involved in polyprotein processing, the NS2/NS3 and the NS3 non-structural proteins that have distinct proteolytic activities. The C-terminal domain of NS2 contains a cysteine PR that constitutes a part of the NS2/NS3 PR which is responsible for the cleavage of the NS2/NS3 junction in a zinc-dependent manner. The autolytic cleavage at this site occurs intramolecularly (*in cis*) [64]. The PR domain of NS3 acts as a serine PR playing a crucial role in the proper folding of NS2/NS3 PR, although, its activity is not required for processing at the NS2/NS3 site [53]. Rather, NS3 catalyses the cleavages at NS3/NS4A, NS4A/NS4B, NS4B/NS5A, and NS5A/NS5B junctions (Fig. 5A). The protease activity of NS3 is complemented *in trans* by the NS4A cofactor that is an amphipathic peptide having hydrophobic N- and hydrophilic C-terminus. A short N-terminal region of NS4A can act as an effector *in trans* even prior to cleavage from the C-terminus of NS3 (Fig. 5B) [44]. The cleavage at NS3/NS4A junction by NS3 was found to occur only *in cis*, while NS4A/NS4B, NS4B/NS5A, and NS5A/NS5B cleavage sites are processed *in trans*. Accordingly, the NS3 PR is released from the polyprotein precursor *via* cleaving itself at its C-terminus intramolecularly [11, 44, 172].

### Enveloped ss(+)RNA viruses - Togaviruses - Alphavirus proteases

Alphaviruses are Group IV viruses that have enveloped ss(+)RNA genome and belong to the *Togaviridae* family (Fig. 1). They are classified into the group of Old- and New-World alphaviruses and are spread worldwide. The group of Old-World alphaviruses includes Chikungunya virus (CHIKV), Sindbis virus (SINV) and Semliki Forest virus (SFV), while the Venezuelan equine encephalitis virus (VEEV), Eastern Equine Encephalitis Virus (EEEV), and Western Equine Encephalitis Virus (WEEV) are New-World alphaviruses. Although, the two groups evolved independently, they share key features of their life cycles such as pathways of polyprotein processing [49].

**Fig. 6:**
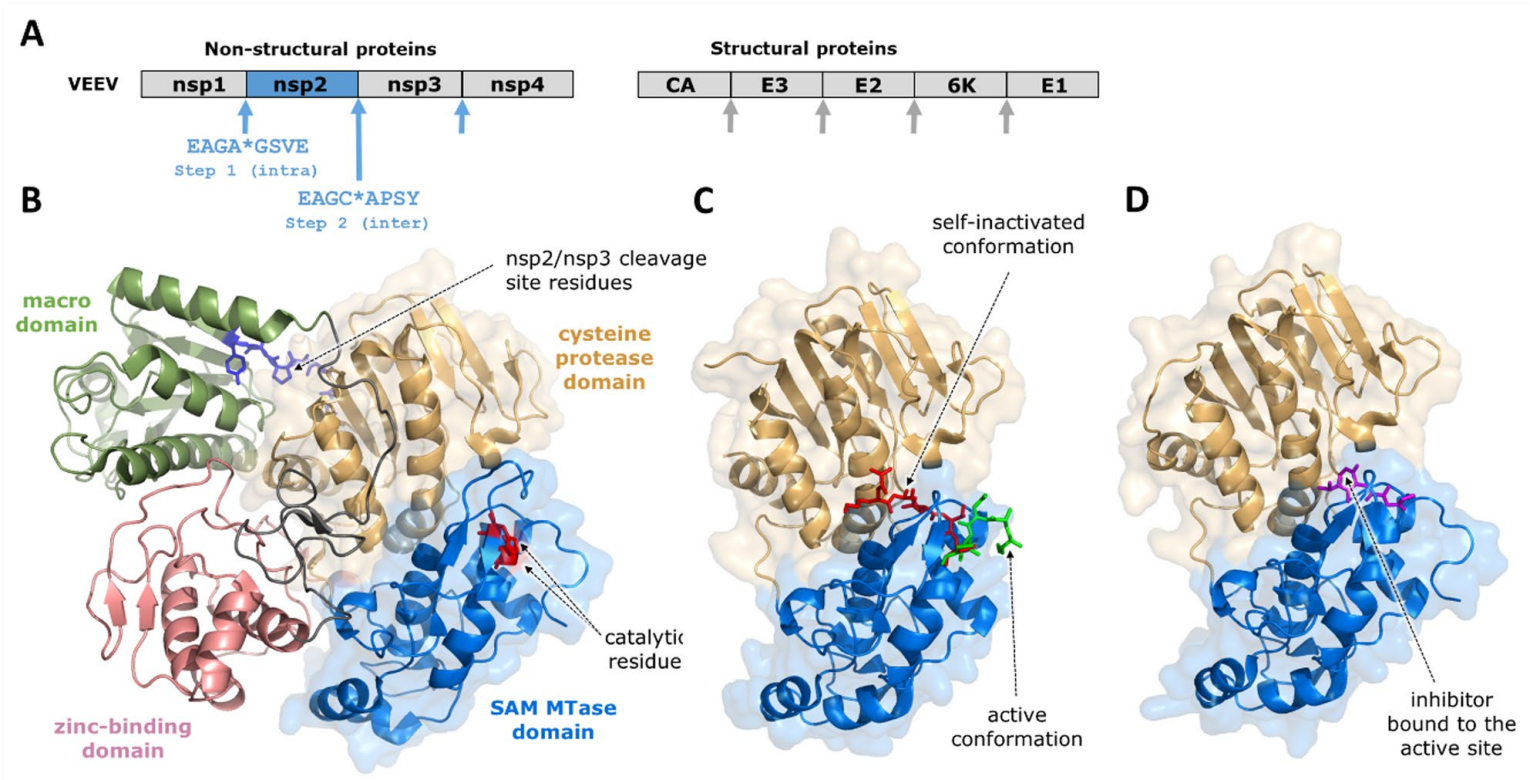
Structure of alphavirus nsP2pro. (**A**) Organization of VEEV polyproteins. The cleavage sites are indicated based on the report of Hernandez et al., [62]. The nsP2pro cleavage sites are shown by blue arrows along with their sequences. Other cleavage sites (capsid, furin, signal peptidase) are indicated by grey arrows. (**B**) Ribbon representation of SINV nsp2/nsp3 precursor (PDB ID: 4GUA) [153]. The N-terminal cysteine protease domain of nsP2pro is shown in light blue and the C-terminal S-adenosyl-L-methionine-dependent RNA methyltransferase domain (SAM MTase) in yellow. The macro domain (green), the zinc-binding domain (pink), and the linker between these domains (grey) are shown. The catalytic Cys and His residues are shown as red sticks, and the P4-P4’ residues of the nspP2/nsp3 cleavage site (GVGA*APSY) as blue sticks. (**C**) The active and self-inactivated conformations of the N-terminus of nsP2pro are also shown (PDB ID: 8DUF) [65]. The N-terminus bound to the active site in the self-inactivated conformation is shown in red, while it is colored green in the active conformation being exposed to the surface. (**D**) Crystal structure of VEEV nsP2pro complexed with a peptide-like inhibitor (PDB ID: 5EZS) [67]. Bound inhibitor to the active site is shown in purple

Genomes of alphaviruses encode structural as well as non-structural polyproteins. The non-structural polyprotein (P1234) consisting of four proteins (nsp1, nsp2, nsp3, and nsp4) (Fig. 6A) is processed by the C-terminal cysteine PR domain of nsp2, referred to as nsP2pro (Fig. 6B) [18]. Studies on the processing of P1234 polyprotein of SINV [58] and SFV [180] revealed that the cleavage at the nsp1/nsp2 junction liberating the N-terminus of nsp2 occurs exclusively *in cis*. The dilution-sensitive nature of the cleavage at the nsp2/nsp3 junction indicated that processing at this site occurs intermolecularly. Structural studies of a model precursor containing the nsp2 PR and the nsp3 central domains of SINV (PDB ID: 4GUA) revealed that the residues of the nsp2/nsp3 cleavage site are distant from the active site of the nsp2’s PR domain (~ 40 Å), suggesting that processing at the C-terminus of nsp2 PR likely occurs *in trans* [153] (Fig. 6). In accordance with this, structural analysis of VEEV nsP2pro also revealed that the nsp2/nsp3 cleavage site is not accessible for intramolecular cleavage, as it is distant from the active site (~ 42 Å) (PDB ID: 2HWK) [145]. These data are in agreement with those of in vitro analyses which revealed processing of nsp2/nsp3 cleavage site *in trans*, both in the case of SINV [58] and SFV PRs [180].

Crystallographic analyses of VEEV nsP2pro helped to visualize the reorganization of the N-terminal residues and their reorganization into the active site using a catalytically inactive mutant (N475A) (PDB ID: 6BCM) [34] and a surface mutant enzyme (K741A and K767A) (PDB ID: 8DUF) [65]. In this conformation the interaction of the N-terminus with the active site (Fig. 6C) was found to mimic the binding of a natural substrate or a peptide-like inhibitor (Fig. 6D). A continuous density observed for the N-terminus of active site- [34] and surface-mutant enzymes [65] implied that binding of these residues to the active site corresponds to a catalytically incompetent conformation. Although the structures representing a self-inactivated state of VEEV nsP2pro do not provide direct evidence for the mechanism of autoactivation *in cis* or *in trans*, the alternate conformations potentially contribute to the regulation of the PR activity [34, 198], and the exact molecular mechanism remains to be elucidated.

Based on the available data, PR precursors of Old- and New-World alphaviruses likely utilize the same mechanism, i.e. processing of the N-terminus of nsp2 containing nsP2pro *in cis* is followed by cleavage of nsp2’s C-terminus *in trans*, resembling the cleavage events of HIV-1 PR and SARS-CoV-2 MPro precursor activation.

### Enveloped ss(+)RNA viruses - Arterivirus proteases

The *Arteriviridae* family belongs to the group of enveloped ss(+)RNA viruses (Fig. 1), employing a polyprotein expression pathway. The prototypic members of this family are the equine arteritis virus (EAV) as well as the porcine reproductive and respiratory syndrome virus (PRRSV), being economic concerns for horse- and swine-breeding industries, respectively. Genome of EAV encodes two large polyproteins, pp1a and pp1ab, that are produced by ribosomal frameshifting and are subsequently processed proteolytically into non-structural proteins by virus-encoded PRs [8]. Of the non-structural proteins of EAV pp1ab (nsp1-nsp12) there are three distinct PR domains: the non-structural protein 1 and 2 (nsp1 and nsp2, respectively) are papain-like cysteine PRs, while nsp4 is a chymotrypsin-like serine PR being considered as the main PR of EAV [156].

Major and minor polyprotein processing pathways have also been established for EAV. Cleavages of the major pathway correspond to highly efficient downstream processing events mediated by nsp4, which release the mature non-structural proteins [9, 46, 186, ]. In this pathway, cleavage of nsp4 occurs first at its C-terminus, the resulting nsp3-4 protein is subsequently cleaved (at its N-terminus) to release the free nsp4 [155]. The nsp2 was found to act as a cofactor of nsp4 PR in the major pathway, it is required for processing at nsp4/nsp5 junction, otherwise cleavage does not occur and further processing events occur *via* the minor pathway [186]. In the minor pathway, nsp4 is cleaved first at its N-terminus (at the nsp3/nsp4 junction) with low efficiency producing a transient or partially processed intermediate, while the nsp4/nsp5 junction remains uncleaved [9, 186]. Crystallographic analysis implied that EAV nsp4 has alternate conformations which enable processing both N and C-termini *in cis* and contribute to regulating proteolytic activity, e.g. by preventing self-inactivation [9]. Precursor of EAV nsp4 PR is processed *via* two alternative cleavage pathways, nsp4 is cleaved first at its C-terminus in the major proteolytic pathway, and cleavage does not occur at its C-terminus in the minor pathway. Accordingly, the mechanism of PR precursor activation of EAV nsp4 does not resemble those of HIV and SARS-CoV-2 PRs.

### Non-enveloped ss(+)RNA viruses - Picornaviruses

**Fig. 7:**
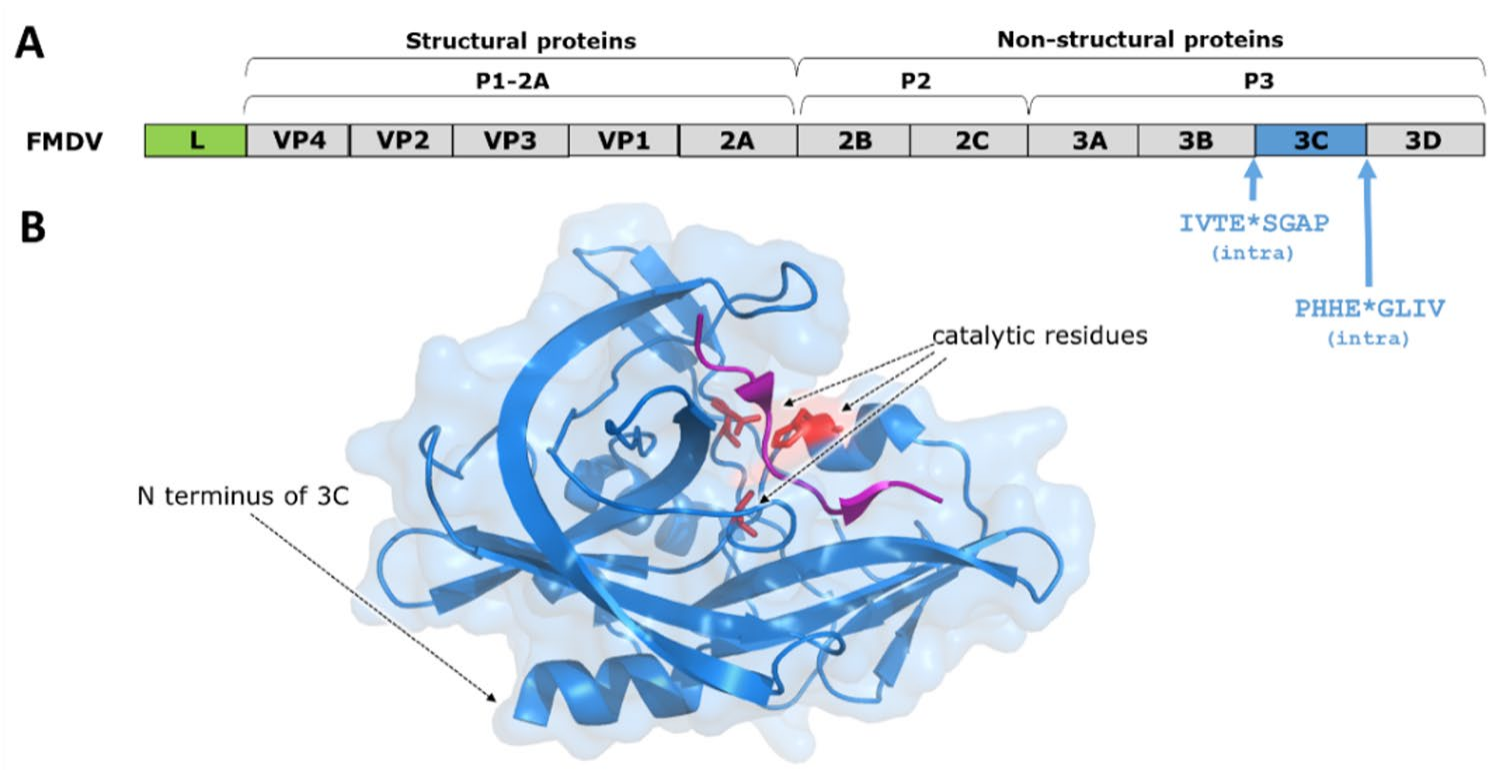
The FMDV polyprotein and the structure of 3 C PR. (**A**) Organization of FMDV polyprotein. N- and C-terminal cleavage site sequences of 3 C PR are indicated [14]. (**B**) Ribbon representation of FMDV 3 C PR (PDB ID: 2WV4) [202]. The enzyme is shown in light blue and the decapeptide that binds to the active site, corresponding to the VP1-2 A cleavage site (APAKQ*LLNFD) in purple. Residues of the Cys-His-Asp catalytic triad are shown as red sticks (the catalytic Cys was mutated to Ala in this structure)

Picornaviruses belong to the group of non-enveloped ss(+)RNA viruses (Fig. 1). The members are classified into multiple genera, such as hepatitis A virus (*Haptovirus* genus), foot-and-mouth disease virus (FMDV) (*Aphthovirus* genus), as well as enteroviruses and rhinoviruses (*Enterovirus genus*) [42]. These viruses also use polyprotein pathway and rely heavily on self-processing PRs to cleave their polyproteins [162], as exemplified by FMDV which infects cloven-hoofed animals and is the causative agent of foot-and-mouth disease [54]. The genome of FMDV contains one open reading frame that encodes a single polyprotein which consists of multiple domains including an autolytic N-terminal protease domain (leader protease, Lpro), capsid proteins (VP1-VP4), a transmembrane protein (3 A), 3B peptides (3B1, 3B2 and 3B3), a protease being similar to chymotrypsin-like serine proteases (3Cpro) and an RNA-dependent RNA polymerase (3Dpol) (Fig. 7A) [76].

Not Lpro but 3Cpro is responsible for the processing of the 2BC-3ABCD polyprotein, it catalyses 10 of the 13 cleavages (Fig. 7A) [36]. FMDV is considered to use alternative mechanism for processing of the P3 precursor, that can be differentially processed intra- and intermolecularly (i.e. *in cis* and *in trans*), which results in the release of alternative precursors (3ABC and 3CD). The two cleavage pathways are mutually exclusive [63, 137]. Studies of picornavirus 3 C PRs revealed that they are cleaved at their N-terminus more readily than at the C-terminus, indicating intramolecular cleavage at the N-terminal cleavage site (*in cis*) [36]. Similar to the cleavage of FMDV 3Cpro at its N-terminus (3B3/3 C junction), processing of the C-terminal cleavage site *(*3 C/3D junction) was also found to occur *in cis* [63]. A switch between the *cis* and *trans*-mediated processing was also reported for FMDV, the mature 3Cpro (or the 3Cpro-containing precursor) was found to mediate P3 processing by *in trans* cleavages, which is thought to contribute to regulation of replication *via* appearance of alternative precursors [63]. Accordingly, activation mechanism of FMDV 3Cpro precursor is likely different from those of HIV-1 and SARS-CoV-2 PRs.

### Non-enveloped ss(+)RNA viruses - Potyviruses

The Tobacco etch virus (TEV) belongs to the *Potyviridae* family of non-enveloped ss(+)RNA viruses (Fig. 1). Polyprotein of TEV consists of 10 protein domains: P1, HC-Pro, P3, 6K1, CI, 6K2, VPg, NIa-Pro, Nlb, and coat protein [1]. Proteolytic processing of the viral polyprotein is mediated by three virus-encoded PRs: (i) the P1-Pro trypsin-like serine PR, (ii) the helper component papain-like cysteine PR (HC-Pro) and (iii) the NIa-Pro serine-like cysteine PR [179]. The functional PR being responsible for polyprotein processing is NIa-Pro that is a 49 kDa protein containing a 27 kDa catalytic domain. It is important to note that NIa-Pro is an indispensable tool for recombinant protein technology, it is one of the most widely applied tool of enzymatic fusion tag removal [51, 78, 116, 187].

During processing of TEV polyprotein, both P1-Pro and HC-Pro cleave themselves at their C-termini *in cis* [55], while all other seven sites are processed by NIa-Pro. NIa-Pro was found to exhibit both intra- and intermolecular cleavage activities. Activity assays performed by using wild-type and cleavage site mutant proteins revealed that the release of NIa-Pro from the precursor is carried out by *in cis* cleavages, without the requirement for a specific order of cleavages at its N and C-termini, while the other sites of the polyprotein are processed *in trans* [22, 23]. Not only self-proteolysis, but also the cleavage of plant host proteins is carried out by NIa-Pro [195]. NIa-Pro of pepper vein banding virus (PVBV) is also cleaved at its termini only *in cis* [146], indicating that the mechanism of PR precursor processing of TEV and PVBV is different from those of HIV-1 and SARS-CoV-2.

### Non-enveloped ss(+)RNA viruses - Caliciviridae - Noroviruses

Noroviruses are non-enveloped ss(+)RNA viruses and belong to the *Caliciviridae* family (Fig. 1). Their genome encodes a chymotrypsin-like 3 C protease, corresponding to non-structural protein 6 (NS6) that is responsible for the processing of the viral polyprotein [21, 43, 59, 159, ]. The model of norovirus (NV) polyprotein processing has already been established based on in vitro assays and by studying cleavage site mutants [12, 149]. The NS1/2-NS3-NS4-NS5-NS6-NS7 viral polyprotein (also referred to as Nterm-NTPase-p20-VPg-Pro-Pol) was found to be processed by “early” cleavages to release the NS1/2 domains. Then, the released NS3-7 precursor is subsequently cleaved by NS6 *via* more efficient “late” cleavages which yields the NS6-NS7 (i.e. Pro-Pol) precursor containing the PR domain. This cleavage occurs at the N-terminus of NS6 intramolecularly (*in cis*). In agreement with this, the extended conformation of the PR domain was assumed to enable the NS5/NS6 (i.e. VPg6Pro) junction to access the active site, making an intramolecular cleavage possible [158]. Autoproteolytic activity was not observed for the NS6-NS7 complex, this precursor is processed further only *in trans*, yielding the NS6 and NS7 proteins. Accordingly, the processing of NV PR precursor resembles that of HIV-1 PRs, namely the NV NS6 is also cleaved at its N and C-termini *via* intra- and intermolecular cleavages, respectively. In contrast to HIV-1 PR, the NS6-NS7 NV precursor has higher proteolytic activity than the free NS6 PR. In addition, the “late” cleavage events were found to occur in two possible pathways where a NS4-NS5-NS6 precursor is also formed, in this case the NS6 is cleaved first at its C-terminus [12]. The existence of different cleavage pathways is thought to contribute to the regulation of the viral replication [21].

### Non-enveloped dsRNA viruses - Birnaviruses

**Fig. 8:**
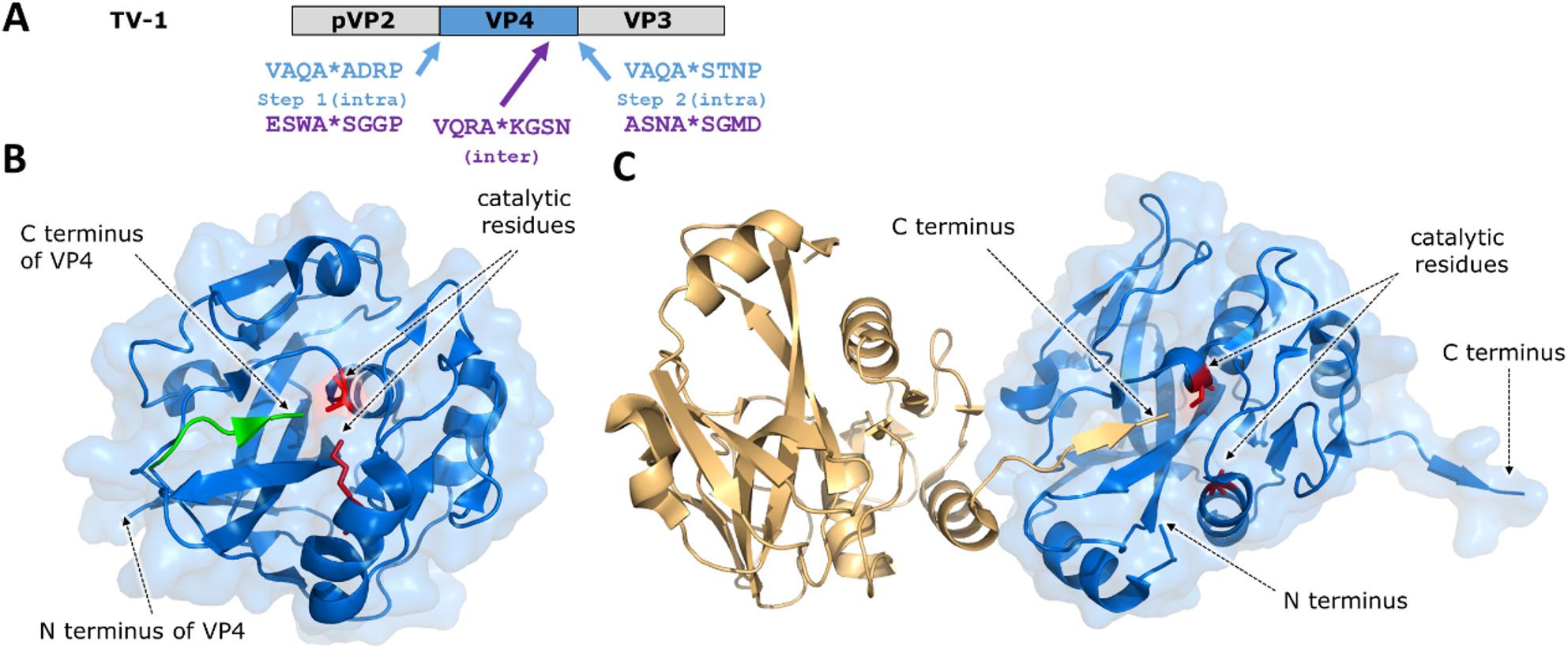
VP4 protease of birnavirus TV-1 and IPNV. (**A**) Organization of TV-1 polyprotein. The VP4 PR domain and sites of self-cleavage of TV-1 VP4 are shown in blue [31]. In addition to terminal sites, an internal cleavage site sequence is also shown for IPNV VP4 (VQRA*KGSN), IPNV sites are shown in purple [94]. (**B**) Ribbon model of TV-1 VP4 (PDB ID: 3P06) [31]. VP4 is blue, its C-terminal residues bound to the active site are green, while the catalytic residues (sticks) are red. (**C**) Intermolecular contacts between two (light orange and blue) active site mutant (Lys-to-Ala) VP4 PRs of IPNV (PDB ID: 2PNL) [94]

Birnaviruses belong to the group of non-enveloped ss(+)RNA viruses (Fig. 1), the *Birnaviridae* family includes infectious bursal disease virus (IBDV), infectious pancreatic necrosis virus (IPNV), Blotched Snakehead virus (BSNV), Drosophila X and B virus (DXV and DBV, respectively), and Tellina virus 1 (TV-1). These viruses use polyprotein pathway for their replication, the viral genome encodes a non-canonical serine-lysine PR referred to as VP4 (Fig. 8A). The investigation of TV-1 revealed that that VP4 PR also undergoes self-proteolysis and TV-1 polyprotein is processed at pVP2/VP4 and VP4/VP3 junctions to release VP4 PR [31, 38].

Neither targeted structural studies nor biochemical assays were performed to study processing of VP4 at its N-terminus, therefore, there are only indirect evidences for the intramolecular cleavage at the VP2/VP4 junction [38]. In contrast, cleavage at the C-terminus of TV-1 VP4 has been studied in a more detailed manner. A crystallographic analysis of TV-1 VP4 showed the binding of the C-terminus to its own active site (Fig. 8B), trapping the acyl-enzyme helped to visualize a cleavage event at VP4/VP3 junction that occurs intramolecularly (*in cis*) [31]. It is noteworthy that VP4 of IPNV was found to exhibit *in trans* activity, as well. The primary processing of the large IPNV polyprotein occurs in *in cis*, the yielded pre-VP2 capsid precursor (pVP2) is subsequently processed by VP4 at multiple sites *via* bimolecular reactions, i.e. conversion of pVP2 to VP2 occurs *in trans* [132]. In addition, IPNV VP4 was found to undergo cleavage at an internal site, as well, where cleavage also occurs *in trans*. The crystal structure of IPNV VP4 revealed the formation of an acyl-enzyme complex between the residues of the internal cleavage site and the active site of the adjacent enzyme, alluding to an intermolecular cleavage event (Fig. 8C) [94].

Although, self-processing at the internal cleavage site *in trans* has been described for IPNV VP4, the activation mechanism of the PR precursor is not identical with those of HIV-1 and SARS-CoV-2, because self-cleavages at the N- and C-terminal boundaries of birnavirus VP4 likely occur only *in cis*.

## Inhibition of protease precursor processing

### Autoprocessing as target for antiviral therapies

The proteolytic processing of the viral polyprotein is indispensable for the maturation of viral proteins, therefore, the PR(s) encoded by the viral genomes are considered as crucial targets of antiviral therapies. The identification and rational design of broad-spectrum PIs is of a special interest [7, 84]. Since PIs of mature PRs are well-reviewed in the literature [16, 56, 110, 201], therefore, in this review only the PIs targeting PR precursors are discussed.

Targeting the precursor with high affinity binders rather than mature PRs may offer considerable advantages for inhibiting the viral replication cycle. Inhibiting the early stages of the life-cycle can reduce the risk of resistance mutations, while the accumulation of uncleaved precursors may trans-dominantly inhibit the production of mature virions [72, 110, 111, 124, ]. For example, PIs of the precursor forms of HIV-1 PR may potentially prevent the stabilization of PR dimers with mature trans-activity [71, 101, 127]. No such PIs that can specifically target the PR precursors have yet been approved for retrovirus (such as HIV-1 PR) [70], coronavirus (such as SARS-CoV-2 MPro), or alphavirus PRs (e.g. VEEV nsP2pro).

From a therapeutic standpoint, the picornavirus PRs, especially 3Cpro, have been thoroughly explored as antiviral targets. Protease inhibitors (PIs) such as rupintrivir (a peptidomimetic inhibitor of rhinovirus 3Cpro) show potent activity against human rhinoviruses by blocking 3Cpro’s cleavage activity, including its autocatalytic maturation [41]. However, due to rapid mutational selection and PR flexibility it has limited clinical use [61, 130].

Clinical high affinity HIV-1 PIs (indinavir, ritonavir, amprenavir, nelfinavir, tipranavir, saquinavir, atazanavir, lopinavir, and darunavir) inhibit both HIV-1 and HIV-2 PR, with variable efficacies [20, 108]. These small molecular inhibitors bind tightly to the active sites of HIV PRs and can inhibit the mature, free enzymes. The inhibition of a mature PR is much more efficient (even ~ 10,000-fold higher) compared to the immature PR that is embedded within the polyprotein [69, 73, 101, 127, 134]. The clinical PIs are unable to inhibit HIV-1 precursor processing in a therapeutically relevant concentration, which is considered to contribute to their failure in the clinic. Darunavir is the most potent inhibitor of HIV-1 PR precursor, due to its ability to engage a secondary binding site within the enzyme, but it is effective only in the micromolar range, and its efficacy is drastically reduced by resistance mutations [101, 127]. Interestingly, an experimental compound (brecanavir) was found to block the processing of the HIV-1 PR precursor more efficiently than the FDA-approved PIs [73], although further investigation is required.

HIV-1 PR is active as a homodimer, each monomer contributing an aspartate to the active site. The dimer upon its release at its termini is stabilized in part by the interactions of antiparallel N- and C-terminal β-strands at the dimer interface, the C-terminal strands forming the interior of the four-stranded β-sheet. The peptides mimicking the interface strands can replace the terminal strands of HIV PR, impairing dimer formation and catalytic activity. Such highly PR-specific peptides are considered as candidate PIs [188] and have already been designed for HIV-1 PR [5, 17, 37, 150, 203]. For example, the HIV-1 PR dimerization inhibitor P27 peptide consists of 27 residues (PQITLRKKRRQRRRPPQVSFNFCTLNF) encompassing the five N- and five C-terminal residues of HIV-1 PR (PQITL and CTLNF, respectively) and a 13 residue-long sequence of HIV-1 Tat’s cell permeable domain (RKKRRQRRRPPQV). These terminal residues are required for the dimerization of HIV-1 PR, which from the antiparallel β-strands of the dimer interface. The P27 peptide was found to inhibit both HIV-1 and HIV-2 PR with IC_50_’s in the low micromolar range [37].

Inhibitors targeting other viral proteins can also influence PR activation. Efavirenz, a non-nucleoside reverse transcriptase inhibitor (NNRTIs) of HIV was found to enhance the dimerization of RT. Since RT and PR domains are part of the Gag-Pol polyprotein, increased RT dimerization may promote polyprotein dimerization, which in turn facilitates PR dimerization and causes premature activation. The premature autoprocessing was found to decrease the production of viral particles [77, 165]. It is important to note that this effect was observed in tissue culture experiments only at micromolar concentration range, but such a high concentration is not relevant from the viewpoint of in vivo therapy. Nevertheless, this highlights the potential of drugs that might cause premature activation of the PR.

HIV-1 PR remains inactive prior to budding, but some NNRTIs such as efavirenz and rilpivirine can cause its premature activation due to enhanced multimerization of Gag-Pol [47], as described above. It has been reported that caspase recruitment domain-containing protein 8 (CARD8) can be cleaved by HIV-1 PR. Premature intracellular activity of HIV-1 PR can be sensed by CARD8 that mediates inflammasome activation, causing pyroptosis of HIV-1-infected cells and clearance of latent HIV-1 in patient CD4^+^ T cells [185]. Accordingly, the use of molecules that can promote Gag-Pol dimerization (and thus causing premature PR activation) is considered as a promising strategy to activate CARD8 inflammasome [85]. In addition, sensitization of CARD8 can improve the efficacy of NNRTI-induced inflammasome activation, enhancing the clearance of HIV-1 from CD4^+^ T cells [32]. The sensitization can be achieved by inhibiting dipeptidyl peptidase 9 (DPP9) that is a negative regulator of CARD8, its binding to CARD8 inhibits inflammasome activation and pyroptosis. The DPP9 inhibitor Val-boroPro (VbP) was found to prevent inhibition of CARD8 by DPP9, increasing the intracellular concentration of CARD8. VbP was found to enhance the efficacy of NNRTIs and can boost CARD8 activity even in the absence of NNRTIs, which might be promising to overcome resistance against NNRTIs via sensitization of CARD8 inflammasome [32].

Similar to HIV-1 PR, the clinically approved SARS-CoV-2 MPro inhibitors nirmatrelvir [93] and ensitrelvir [164] have well-documented efficacy against the mature enzyme. However, the precursor form was found to be less sensitive to inhibition [72, 124]. Unlike HIV-1 PR, dimerization of MPro results in the active site loop equilibrium shifting from an inactive to an active state. It is evident from isothermal titration calorimetric experiments that potent clinical inhibitors [119] and GC373 [Nashed, Kneller et al., 2022] [3] that were designed to bind to the active sites of MPro dimer exhibit very weak binding to the monomer form. Therefore, MPro and enzymes alike, which exhibit an inactive-to-active conformational equilibrium, provide an alternative strategy for drug design targeting the inactive state of the precursor monomer [163, 197]. However, potent inhibitor molecules that can selectively target the precursor SARS-CoV-2 MPro active site or that perturb its dimerization [52, 124] are currently unavailable.

### Targeting viral protease cofactors

Cofactor-dependent activation represents a unique vulnerability in several viral PRs, offering a promising strategy for selective antiviral intervention. Unlike catalytic sites, which often share structural homology with host enzymes and pose challenges for specificity, cofactors and their interaction interfaces tend to be virus-specific. This specificity reduces the risk of off-target effects and opens new possibilities for therapeutic targeting. As mentioned previously, disrupting the interaction between NS2B and NS3 in the case of flavivirus PRs (such as DENV and ZIKV) has already yielded potent inhibitors that either prevent complex formation or allosterically stabilize an inactive conformation [161]. These approaches underscore the feasibility of targeting cofactor dynamics as a viable drug strategy. Similarly, viral DNA and the pVIc peptide are both crucial cofactors for adenovirus PR activation. Targeting these interactions could inhibit virion maturation without affecting host PRs. For instance, molecules that mimic pVIc but fail to promote proper folding could act as decoy inhibitors, while intercalators or groove-binders could obstruct DNA-mediated activation [113].

Other cofactor-dependent systems, such as HCV NS3 which requires NS4A (Fig. 5B) [44], the NS3 catalytic domain of flaviviruses that utilize NS2B peptide as cofactor (Fig. 4B) [45], and herpes viral PRs, which often depend on scaffold proteins or dimerization [152], further highlight the diversity of viral cofactor mechanisms. Each presents a potential site for pharmacological intervention, particularly where the cofactor contributes to structural stabilization or substrate orientation. As our understanding of these protease–cofactor interactions deepen, rational drug design can increasingly exploit these dependencies, thereby offering a pathway to develop broad-spectrum or virus-specific antivirals with an improved drug resistance safety profile.

## Functional characteristics of proteases

### Cleavage site sequences

The PRs (or the non-structural proteins encompassing the PR domain) that release themselves from the polyprotein contain consensus cleavage site sequences at their N and C-termini, being specific for the given PR. For example, SARS-CoV-2 MPro shows high specificity for the cleavage sites containing leucine in P2, glutamine in P1, and Ser/Ala/Gly residue in P1’ position [90]. Both its N- and C-terminal self-cleavage sites correspond to the consensus sequence to facilitate MPro autoprocessing. Likewise, the self-processing sites of SARS-CoV-2 PLpro also represent its own consensus cleavage site sequence (Fig. 3A). Interestingly, unlike MPro and PLpro, natural cleavage sites of HIV-1 PR at its termini do not have a consensus sequence motif (Fig. 2A). Rather, their sequences are diverse, the recognition is strongly sequence context-dependent [174, 175] and determined by the shape and space filled by the substrate [138]. This is referred to as substrate envelope model or hypothesis that has been defined for SARS-CoV-2 MPro, as well [151]. The N- and C-terminal cleavage sites of ZIKV NS3 protease (NS2B/NS3 and NS3/NS4A, respectively) are also highly similar and represent the consensus recognition sequence of the PR (Fig. 4A) [154, 183], similar to the nsp1/nsp2 and nsp2/nsp3 cleavage sites of VEEV (Fig. 6A) [115]. Accordingly, the cleavage sites that are processed *in cis* do not have strikingly different sequence as compared to the sites that are cleaved *in trans*; and the presence of the consensus cleavage sites at the termini of a given PR ensures its self-release from the polyprotein by *in cis* and/or *in trans* cleavages.

The cleavage sites at N and C-termini of the PR domain might be considerably different if more than one PR facilitates polyprotein processing. In these cases, different cleavage site sequences are recognized by these PRs. For example, genome of HCV encodes two different PRs which release the NS3 PR from the polyprotein: the NS3 protein is cleaved at its N-terminus by the NS2/NS3 zinc-dependent protease domain at the NS2B/NS3 cleavage site, while the NS3/NS4 junction at the C-terminus is cleaved by NS3 [44]. Accordingly, the cleavage site sequences at the termini of HCV NS3 non-structural protein represent a different consensus sequence (Fig. 5A).

The cleavage site sequences do not provide direct information about the order of self-cleavages, and the sequential order cannot be estimated based on cleavage efficiencies of substrates representing different autolytic sites because the enzyme kinetic measurements and specificity studies are performed almost exclusively in the context of the mature enzymes, and the oligopeptide substrates that represent the intramolecular cleavage sites can be easily processed in the in vitro assays by the mature enzymes *in trans*.

### Evolution and functional significance of proteases

The ability of viral PRs catalysing their release from their polyprotein precursors is a conserved pathway across numerous viral families, serving multiple evolutionary and functional purposes. The long polyproteins, which are post-translationally processed into individual functional units, reduce the need for complex gene regulation strategies such as multiple promoters or splicing events. Particularly in ss(+)RNA viruses like picornaviruses, flaviviruses, and coronaviruses, self-cleaving PRs not only initiate their own release but also coordinate the maturation of viral proteins in precise amounts. Functionally, self-proteolysis enables temporal and spatial regulation of viral protein activation, ensuring that PRs are activated when meeting the requirements of transient correctly folded (*in cis* cleavage), and subsequently capable of initiating further processing steps (*via* delayed *in trans* cleavage), thereby avoiding premature cleavage events that could disrupt replication or assembly. Additionally, this autonomous activation pathway reduces reliance on host PRs, and especially advantageous in diverse cellular environments. For example, SARS-CoV-2 MPro self-activates within the infected cell to coordinate the replication cycle [144], while in retroviruses like HIV, the activation of the PR is delayed until after particle assembly, ensuring infectivity only after proper assembly [166]. The order of Gag polyprotein cleavage is also modulated through conformational dynamics within the flaps of HIV PR leading to transient, sparsely populated production complex between PR and Gag substrates [39]. The modular nature of self-cleaving PRs also supports evolutionary plasticity, allowing cleavage sites and domains to adapt independently to new substrates or host conditions, as observed in 3Cpro and NS3 PRs [112, 129, 177]. Moreover, many viral PRs acquire multi-functionality after self-processing, engaging in immune evasion by targeting host signaling proteins such as mitochondrial antiviral-signaling protein (MAVS) or Toll-like receptor 3 adaptor protein (TRIF) [Chin et al., 2022]. Thus, self-proteolysis not only enhances viral replication efficiency, but also underscores the protease’s broader role in host-virus interactions and evolutionary adaptation.

## Summary

Most of the RNA viruses utilize the polyprotein synthesis pathway for replication, and the large polyprotein(s) translated from the viral genome are cleaved subsequently into functional proteins. In contrast, most ss(-)RNA viruses do not use this pathway as they have a segmented RNA genome. The dsRNA viruses exhibit differences in their replication. The enveloped dsRNA viruses (such as *reoviridae*) have segmented genome, while the genome of non-enveloped dsRNA viruses is non-segmented and they use polyprotein s pathway (such as birna- and orthototiviruses). Accordingly, the replication of the non-enveloped dsRNA viruses is more similar to those of ss(+)RNA viruses.

**Fig. 9:**
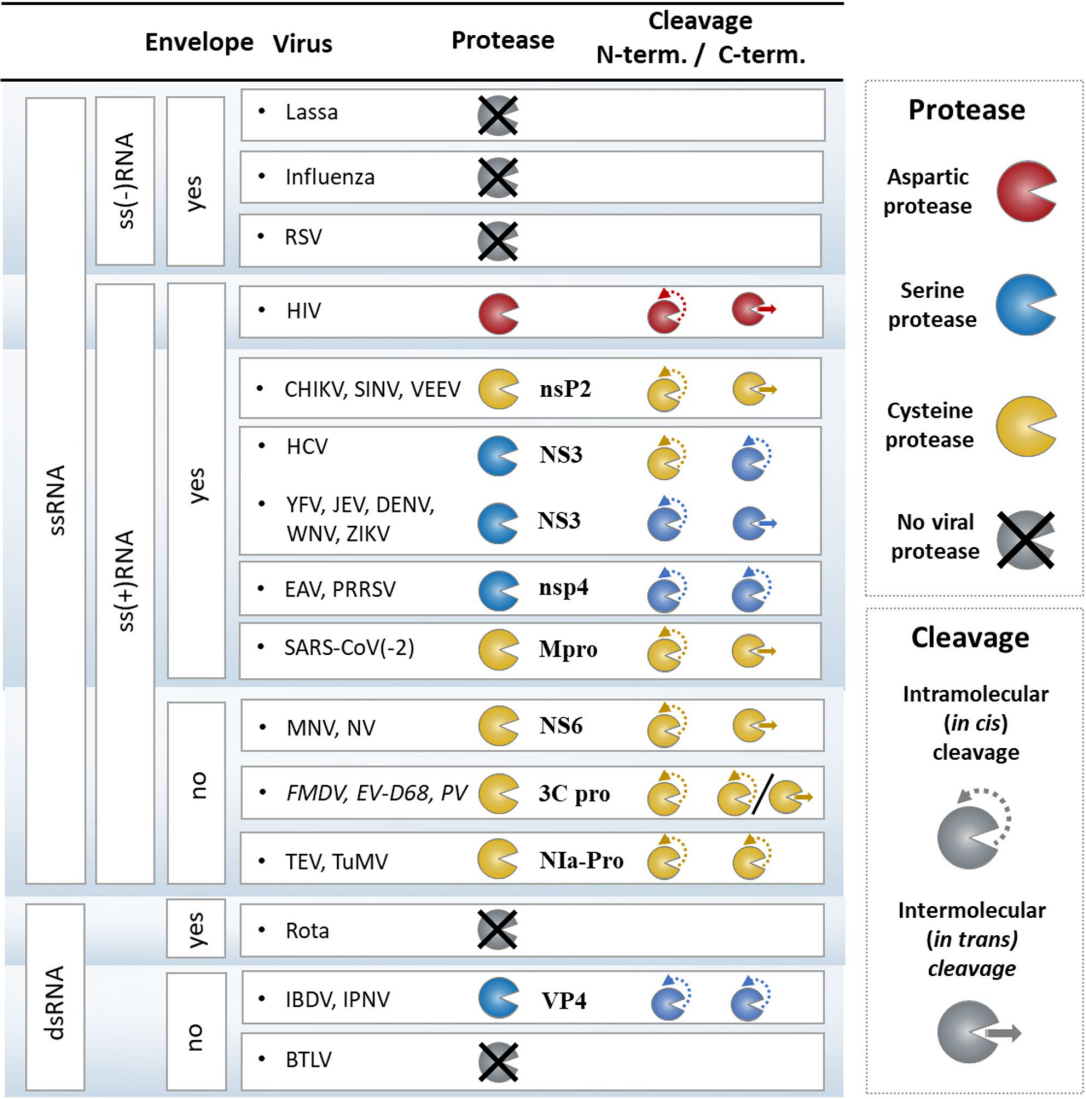
Protease precursor activation of RNA viruses. The RNA viruses are grouped based on their genome and replication strategy (Baltimore classification). Salient families of the groups and medically important viruses are listed. The viral PRs are colored based on their catalytic mechanism. The symbols indicate the type of cleavage (intra- or intermolecular) at the N and C-terminus of the protease during PR precursor processing. Question mark indicates yet unknown mechanism

The viral polyproteins can be processed by virus-encoded and/or by host PRs. The ss(-)RNA viruses having segmented genome do not have their own PR, while the genomes of most ss(+)RNA viruses using polyprotein pathway encode at least one PR. Most of the dsRNA viruses also lack a viral PR, nevertheless, birnaviruses have their own (VP4 PR). The majority of ss(+)RNA virus PRs are serine or cysteine PRs, while only retroviruses encode an aspartic PR. Some of the RNA viruses have a non-canonical PR, for example the VP4 protease of dsRNA birnaviruses contains a Ser-Lys catalytic dyad (Fig. 9). Downregulation of activity through self-proteolysis at specific sites within the PR upon depletion of natural substrates is also a feature observed in HIV PRs [107, 114].

Viruses that encode their own PR(s) exhibit differences in polyprotein processing, especially in the mechanism of PR precursor activation. This includes the *in cis* or *in trans* pathways and sequential order of the cleavages that vary between these viruses (Fig. 9). Despite these differences, all the mature PRs retain their trans-activity upon their release. Polyproteins of HIV-1 and SARS-CoV-2 are processed exclusively by the viral PR. Not only viral but host PRs might also contribute to polyprotein processing, such as in the case of ZIKV and HCV. Some of the viruses use only intramolecular cleavages to release the PR from the precursor, such as ss(+)RNA potyvirus TEV and picornavirus FMDV, as well as dsRNA birnavirus VP4 PR.

The PRs of enveloped ss(+)RNA viruses such as coronaviruses (e.g. SARS-CoV-2), flaviviruses (e.g. ZIKV), and alphaviruses (e.g. SINV) share the PR precursor activation mechanism of HIV-1 PR. In these viruses, the intramolecular (*in cis*) cleavage at the N-terminus of the PR is followed by intermolecular (*in trans*) processing of its C-terminus (Fig. 9). This self-activation mechanism is utilized by most enveloped ss(+)RNA viruses either replicating with (like retroviruses) or without DNA intermediate (Fig. 9). It is important to note that the number of viruses whose PR precursor activation has been explored in detail is only limited, accordingly, it is hard to establish general activation mechanism for all RNA virus groups and families. For example, the molecular mechanism of PR precursor processing has not been determined for all retroviruses but only for HIV-1 PR. Consequently, it can be only hypothesized that similar to HIV-1 PR, PRs of other retroviruses (e.g. human T-lymphotropic virus, HTLV) are also cleaved first at their N-terminus intramolecularly, followed by intermolecular processing at their C-terminus.

In order to determine possible determinants of PR precursor activation mechanism, we correlated some characteristics of the viral life-cycles with those of the activation mechanism. Utilization of only intramolecular cleavages (e.g. flavivirus HCV, arterivirus EAV, potyvirus TEV, and picornavirus FMDV PRs), was found to be characteristic for both enveloped and non-enveloped RNA viruses (Fig. 9). Only monomeric enzymes are processed at their N- and C-termini *in cis*, but this is not a unique feature because other monomeric PRs are also processed *in trans*, such as calicivirus NV and togavirus CHIKV PRs. The catalytic mechanism can not be considered as a sole determinant of the utilized mechanism because *cis*-only activation was reported for both serine and cysteine PRs. In addition, the use of a cofactor is not a prerequisite for *cis*-only activation, as e.g. TEV PR does not use any cofactor.

Besides their distinct kinetic behaviours (the *in cis* cleavages are dilution-insensitive), the intra- and intermolecular cleavage reactions exhibit other considerable differences. The *in cis* cleavages are unimolecular and therefore, such cleavages are independent from the stoichiometry of the enzyme and the “substrate” (i.e. the PR being a part of the polyprotein). The *in cis* cleavages can be carried out efficiently even at low precursor concentration and do not require maturation or oligomerization of the PR (except HIV PRs that are active as obligate homodimers). In addition, the precursor forms are less susceptible for active site-directed inhibition than the mature enzymes. Based on our comparison, using both *in cis* and *in trans* cleavages is a more common activation mechanism among the RNA viruses (Fig. 9). This activation mechanism is considered to enable a faster activation of PR precursors, due to the amplification. Namely, a single PR can activate multiple other PRs through *in trans* cleavages, making the precursor activation more rapid (as a positive feedback), while the intramolecular activation is limited to the precursor itself and no amplification occurs. In addition, the *in cis*-processing is considered to be a rate-limiting cleavage step of precursor activation that occurs much slower than the subsequent *in trans* cleavages (such as the activation of HIV and SARS-CoV-2 PRs).

Processing of viral PR precursors is a rate-limiting step of virus maturation, therefore, inhibitors that can block the precursor forms of the proteases are considered to be promising targets of drug discovery, and targeting immutable components of the cells such as the inflammasome, causing premature PR activation, is also a promising approach. In addition, targeting the interactions between the PRs and their cofactors is also a possible approach to inhibit PR maturation, emphasizing the significance of understanding precursor processing. The presence of a complete active site in SARS-CoV-2 MPro and enzymes alike, unlike that of HIV PR in which the active site is formed upon dimerization, underscores the importance of identifying and characterizing compounds that tightly bind to the inactive state of the PR precursor and thereby providing an alternative avenue to arrest polyprotein maturation. The latter strategy may be advantageous in curbing drug resistance invariably observed upon treatment with drugs designed to bind to mature PRs.

## Data Availability

No datasets were generated or analysed during the current study.

## Abbreviations

AIDS: Acquired immunodeficiency syndrome
BSNV: Blotched Snakehead virus
BTLV: Birch toti-like virus
CHIKV: Chikungunya virus
3CLpro: 3-chymotrypsin-like protease
DBV: Drosophila B virus
DENV: Dengue virus
DXV: Drosophila X virus
EAV: Equine arteritis virus
EEEV: Eastern Equine Encephalitis Virus
EV: Enterovirus
FMDV: Foot-and-mouth disease virus
HCV: Hepatitis C virus
HIV-1: Human immunodeficiency virus type 1
HIV-2: Human immunodeficiency virus type 2
HTLV: Human T-lymphotropic virus
IBDV: Infectious bursal disease virus
IPNV: Infectious pancreatic necrosis virus
MERS: Middle East respiratory syndrome coronavirus
MNV: Murine norovirus
MPro: Main protease
NNRTI: Non-nucleoside reverse transcriptase inhibitor
Nsp: Non-structural protein
NV: Norovirus
PI: Protease inhibitor
PLpro: Papain-like protease
PRRSV: Porcine reproductive and respiratory syndrome virus
PV: Picornavirus
PVBV: Pepper vein banding virus
PR: Protease
RSV: Respiratory syncytial virus
RT: Reverse transcriptase
SARS-CoV: Severe acute respiratory syndrome coronavirus
SARS-CoV-2: Severe acute respiratory syndrome coronavirus 2
SFV: Semliki Forest virus
SINV: Sindbis virus
TEV: Tobacco etch virus
TFP: Trans-frame region (TFR) peptide
TFR: Trans-frame region
TV-1: Tellina virus 1
VEEV: Venezuelan equine encephalitis virus
WEEV: Western equine encephalitis virus
WNV: West Nile virus
YFV: Yellow fever virus
ZIKV: Zika virus
